# Disentangling the value equation: a step forward in value-based healthcare

**DOI:** 10.1093/eurpub/ckae060

**Published:** 2024-06-14

**Authors:** Borja García-Lorenzo, Itxaso Alayo, Arantzazu Arrospide, Ania Gorostiza, Ane Fullaondo, VOICE Study Group, Susana Castelo Zas, Patricia Cobos Baena, Inés Gallego Camiña, Begoña Izaguirre Narbaiza, Gaizka Mallabiabarrena, Iker Ustarroz-Aguirre, Alina Rigabert, William Balzi, Roberta Maltoni, Ilaria Massa, Isabel Álvarez López, Sara Arévalo Lobera, Mónica Esteban, Marta Fernández Calleja, Jenifer Gómez Mediavilla, Manuela Fernández, Manuel del Oro Hitar, María del Carmen Ortega Torres, María Consuelo Sanz Ferrandez, Luís Manso Sánchez, Pablo Serrano Balazote, Carolina Varela Rodríguez, Mario Campone, Sophie Le Lann, Piet Vercauter, Kurt Tournoy, Marina Borges, Ana Sofía Oliveira, Marta Soares, Iratxe Vázquez Lerma, Izaskun Artola Irazabal, Aitor Fernandez de Larrinoa Santamaría, Andere Frias Capanaga, Eduardo Vicario Elorduy, Teresa Acaiturri-Ayesta, Elisa Gómez-Inhiesto, Valentina Danesi, Nicola Gentili, Andrea Roncadori, Fátima Hermoso Alarz, Karmele Imaz Iraola, Valerie Adam, Helene De Rijck, Ellen Everaert, Esmeralda Barreira, Pedro Medeiros, Emanuel Barros

**Affiliations:** Biosistemak Institute for Health Systems Research, Torre del Bilbao Exhibition Centre, Barakaldo, Basque Country, Spain; Biosistemak Institute for Health Systems Research, Torre del Bilbao Exhibition Centre, Barakaldo, Basque Country, Spain; Ministry of Health of the Basque Government, Vitoria-Gasteiz, Spain; Biosistemak Institute for Health Systems Research, Torre del Bilbao Exhibition Centre, Barakaldo, Basque Country, Spain; Biosistemak Institute for Health Systems Research, Torre del Bilbao Exhibition Centre, Barakaldo, Basque Country, Spain

## Abstract

**Background:**

The value equation of value-based healthcare (VBHC) as a single figure remains ambiguous, closer to a theoretical framework than a useful tool for decision making. The challenge lies in the way patient-centred outcomes (PCOs) might be combined to produce a single value of the numerator. This paper aims to estimate the weights of PCOs to provide a single figure in the numerator, which ultimately will allow a VBHC figure to be reached.

**Methods:**

A cohort of patients diagnosed with breast cancer (*n* = 690) with a 6-month follow-up recruited in 2019–20 across six European hospitals was used. Patient-reported outcomes (PROs), clinical-related outcomes (CROs), and clinical and socio-demographic variables were collected. The numerator was defined as a composite indicator of the PCOs (CI-PCO), and regression analysis was applied to estimate their weights and consequently arrive at a single figure.

**Results:**

*Pain* showed as the highest weight followed by *physical functioning*, *emotional functioning*, and *ability to work*, and then by a symptom, either *arm* or *breast*. PCOs weights were robust to sensitivity analysis. The CI-PCO value was found to be more informative than the health-related quality of life (HRQoL) value.

**Conclusions:**

To the best of our knowledge, this is the first research to combine the PCOs proposed by ICHOM to provide a single figure in the numerator of the value equation. This figure shows a step forward in VBHC to reach a holistic benchmarking across healthcare centres and a value-based payment. This research might also be applied in other medical conditions as a methodological pathway.

## Introduction

Value-based healthcare (VBHC) suggests that health systems need to be managed in terms of patient-centred outcomes (PCOs).^1^ This approach requires the strategic engagement of healthcare managers and technological advances to collect the PCOs.^2^ VBHC proposes to link the PCOs to costs, and thereby determine the value of the healthcare. In other words, VBHC aims to assess the healthcare outcomes achieved per monetary unit spent.

Various authors have tried to define the concept of value, detail the outcomes of interest for a variety of diseases, or outline a theoretical framework for the best implementation approach.^2–4^ A standardized equation for value would help to benchmark health services in a specific disease in terms of value. The well-known value equation for the VBHC determination is that introduced by Porter et al. in 2010^3^, where VBHC is defined as the ratio of PCOs divided by the costs to achieve those outcomes. However, the definition of the value of healthcare remains ambiguous, closer to a theoretical framework than a useful tool for decision making. In the current theoretical framework, it is not straightforward to assess healthcare in terms of value when value is defined by a series of indicators. The joint interpretation of PCOs has been claimed previously to be a primary challenge in VBHC.^5^ Therefore, it is necessary to establish not only a standard outcome set but also the contribution of each outcome set in the calculation of a single value figure, as a synthetized indicator.

The VOICE community (Value-based Healthcare for Outcomes in Breast and Lung Cancer in Europe), a cluster of eight European healthcare centres, intends to move towards a VBHC delivery system addressing the VBHC in breast and lung cancer from theory into practice. In this context, this paper aims to estimate the contribution of the aforementioned PCOs to provide a single figure in the numerator of the value equation, and therefore a single figure for VBHC, in the context of breast cancer. However, this research might also be useful for other medical conditions as a methodological pathway.

## Methods

### Study design

Data from the breast cancer cohort of the VOICE study was used. This study was designed as a prospective multicentre cohort across six pilot sites in three countries: *Organización Sanitaria Integrada Ezkerraldea-Enkarterri-Cruces*, *Organización Sanitaria Integrada Donostialdea*, *Hospital Juan Ramón Jiménez*, and *Hospital Universitario 12 de Octubre* (Spain), *Istituto di Ricovero e Cura a Carattere Scientifico (IRCCS): Istituto Romagnolo per lo Studio dei Tumori (IRST) ‘Dino Amadori’* (Italy), and *Institut de Cancérologie de l'Ouest* (France), with the approval of the Basque Country Ethics Committee (PI2018107).

The study population consists of patients diagnosed with early breast cancer between 2019 and 2020, meeting the following eligibility criteria: (i) aged over 18 years, (ii) female, (iii) newly diagnosed invasive breast cancer (stage I–III) or ductal carcinoma *in situ* (DCIS), and (iv) with any treatment (surgery, radiotherapy, chemotherapy, hormone therapy, and/or targeted therapy). Criteria for exclusion included patients with (i) rare tumours, (ii) lobular carcinoma *in situ*, or (iii) metastatic disease. Candidates were progressively recruited by their healthcare professionals at diagnosis during medical/nursing visits and provided with the VOICE community information. Participating patients signed an informed consent and were followed for 6 months after diagnosis or until death, whichever came first.

### Data collection

Socio-demographic variables, patient-reported outcomes (PROs), and clinical-related outcomes (CROs) were collected according to the International Consortium for Health Outcomes Measurements (ICHOM) standard set^6^^,^^7^; socio-demographic variables were collected at diagnosis. PRO and CRO information was collected at baseline and followed up at 6 months. HRQoL was collected using the instrument EQ-5D-3L^8^ for the *Organización Sanitaria Integrada Ezkerraldea-Enkarterri-Cruces* (40% of the cohort), and was mapped^9^^,^^10^ from the EORTC QLQ-C30^11^ instrument for the rest of the healthcare centres. In order to ensure that there was no intention to re-identify individuals, data processing agreements were bilaterally formalized between the data processor and each data provider (six healthcare centres) and patient data used in the analyses were anonymized.

### Descriptive analysis

A descriptive analysis of clinical and socio-demographic variables across sites was carried out, followed by chi-square statistical tests to assess their statistical differences.

### Composite indicators

The usefulness of composite indicators has been increasingly recognized for the analysis of complex, multidimensional issues.^12^ Given the framework of PCOs to be considered in the numerator of the value equation^3^ followed by the standard sets of PCOs developed by ICHOM,^6^ the numerator figure was conceived as a composite indicator following Schöner et al.^13^ The OCDE^14^ and Nardo et al.^15^ identified different methodological approaches and their corresponding steps to build composite indicators. Schöner et al.^13^ stated that composite indicators might play an important role in managing several PROs. Following the suggestion by Barclay et al.^12^ to develop quantitative methodological approaches and sensitivity analysis, this paper proposes a regression analysis approach followed by sensitivity analysis, with the aim of estimating the weights of the aforementioned PCOs to provide a single figure in the numerator of the value equation, which ultimately should allow a VBHC single figure to be reached. A sensitivity analysis to explore the robustness of results was then conducted.

The methodological pathway worked as follows: (i) the composite index of the PCOs is defined as the weighted average of the PCOs; (ii) the PCOs weights were estimated by running regression models where the PCOs were the independent variables and the HRQoL was the dependent variable, used as proxy of the numerator; (iii) based on the standardized coefficients from the regression models and their statistical significance, coefficients were transformed into weights. Finally, according to the theoretical composite index definition, weights and PCO were used to estimate the composite indicator.

Let the composite indicator of PCO of patient *i* be a composite indicator from the weighted aggregation of the *K* outcomes ${\text{PCO}}_{ki}$ of patient *i* to be included in the numerator of the value equation, Eq. (1):

(1) $${\text{CI}-\text{PCO}}_{i}=\sum_{k=1}^{k\;=\;K}w_{k}{\;\cdot \;\mathrm{PCO}}_{ki},$$

where ${\text{CI}-\text{PCO}}_{i}$ of patient *i* is the weighted sum of the ${\text{PCO}}_{ki}$ of patient *i*, while $w_{k}\;$represents the weight of the *k*th element of the ${\text{PCO}}_{ki}$, being the ${\text{PCO}}_{i}$ defined as the standardized set of ${\mathrm{PRO}}_{i}$ and ${\mathrm{CRO}}_{i}$, patient-reported and clinical-related outcomes, respectively. For equal directionality and an intuitive interpretation, each PCO was rescaled so that a higher value indicates more improvement.

### Regression analysis

A regression model might inform about the relationship between a number of indicators and a single outcome. A regression model was then estimated to retrieve the standardized coefficients for the PCOs in order to reproduce the HRQoL—the well-known, generic, and widely used utility measure to assess healthcare programmes or interventions—used as a proxy of the health outcome to be measured in the numerator of the value equation.^16^ A regression model was controlled for patient heterogeneity through clinical and socio-demographic patient characteristics, and estimated by ordinary least squares as follows in Eq. (2):

(2) $${\mathrm{HRQoL}}_{i}=\alpha_{0}+\sum_{k=1}^{K}\beta_{k}\cdot {\mathrm{PCO}}_{ki}+{\sum_{j=1}^{J}\emptyset_{j}X_{ji}+\mu }_{i},$$

where ${\mathrm{HRQoL}}_{i}$ is defined as the quality of life of patient *i*, covariate ${\mathrm{PCO}}_{ki}$ is defined as the *k*th element of PCOs of patient *i*, and $X_{i}$ as the vector of the control variables *age*, *education*, *menopause*, *comorbidities*, *early tumour stage*, *ES+/PR+ receptor status*, *HER2 receptor status*, and *site* of patient *i.* Both sets of covariates ${\mathrm{PCO}}_{i}$ and $X_{i}$ are defined in the data collection reference guide of ICHOM.^7^  $\alpha_{0}$, $\beta_{k}$, and $\phi_{j}\;$are the constant term and the parameters associated with ${\mathrm{PCO}}_{ki}\;$covariates and $X_{ji}$, respectively, while $\mu_{i}$ is the random error term. Statistical significance and quantitative magnitude of $\beta_{k}^{s}$ standardized parameters are intended to illustrate the weights $w_{k}$ of PCOs on the HRQoL as follows in Eq. (3):

(3) $$w_{k}=\frac{\beta_{k}^{s^{\prime }}}{\sum_{k=1}^{k=K}\beta_{k}^{s^{\prime }}\;}$$

where $\beta_{k}^{s^{\prime }}$is the statistically significant parameter at 5% of significance level of the ${\mathrm{PCO}}_{ki}\;$covariates.

A full model including all potential covariates was first estimated, then a reduced model was estimated using a stepwise regression strategy to eliminate covariates that did not affect the indicator or showed multicollinearity based on the factor inflation variance (FIV). The stepwise criteria was not only based on the significant level of the independent variables but also on their relevance according to the objective, therefore, following a backward-elimination approach, control variables were prioritized to be removed before the PCO. Then, the CI-PCO_*i*_ under regression analysis was computed as in Eq. (1).

### Sensitivity analysis

As stated in the outline of this paper, the regression analysis was conducted exploiting the information at 6 months and in-differences. To explore the results between time structures, the scatterplot and the corresponding Pearson coefficients between the ${\text{CI}-\text{PCO}}_{i}\;$at 6 months and in-differences were calculated. Additionally, the Mann–Whitney *U* test between the estimated weights at 6 months and in-differences was computed to explore significant differences across PCOs weights between periods.

Although using a single measure such as the HRQoL might not enable a composite indicator to be developed,^17^ the VBHC contributes by adding the condition-specific and a patient-centred approach beyond the one single utility measure widely used. To explore the above-mentioned issue, the scatterplot and the corresponding Pearson coefficient between the ${\text{CI}-\text{PCO}}_{i}\;$and the ${\text{HRQoL}}_{i}\;$over both time structures were computed. All analyses were performed using R version 4.2.2, and the statistical significance was set at *P* < 0.05.

## Results

### Descriptive analysis

A total of 690 patients diagnosed between 2018 and 2020 were recruited. The average age was 59 years (SD = 12); 42.3% reported comorbidities and 66% had post-menopausal status. The sample description is shown in Table 1. Of 690 patients, 638 (92.5%) completed all the PROs at baseline, and 616 (96.6%) completed at least one PRO in a 6-month follow-up. Supplementary Table S1 shows the statistical descriptive of the PROs at baseline and at 6 months.

**Table 1:** Descriptive analysis.^a^

	Total	Site A	Site B	Site C	Site D	Site E	Site F	*P*-value^b^
	*N* = 690	*N*_A_ = 273 (39.6%)	*N*_B_ = 58 (8.4%)	*N*_C_ = 42 (6.1%)	*N*_D_ = 99 (14.3%)	*N*_E_ = 127 (18.4%)	*N*_F_ = 91 (13.2%)	
Patient characteristics								
Age at diagnosis (*N* = 690)								<0.001***
≤50	198 (28.7%)	80 (29.3%)	23 (39.7%)	16 (38.1%)	33 (33.3%)	26 (20.5%)	20 (22.0%)	
51–70	348 (50.4%)	152 (55.7%)	22 (37.9%)	24 (57.1%)	40 (40.4%)	65 (51.2%)	45 (49.5%)	
>70	144 (20.9%)	41 (15.0%)	13 (22.4%)	2 (4.76%)	26 (26.3%)	36 (28.3%)	26 (28.6%)	
Educational level (*N* = 573)								<0.001***^c^
None	32 (5.58%)	19 (7.60%)	0 (0.00%)	1 (2.38%)	12 (12.1%)	0 (0.00%)	NA	
Primary	164 (28.6%)	76 (30.4%)	13 (23.6%)	9 (21.4%)	33 (33.3%)	33 (26.0%)	NA	
Secondary	229 (40.0%)	78 (31.2%)	22 (40.0%)	19 (45.2%)	32 (32.3%)	78 (61.4%)	NA	
Tertiary	148 (25.8%)	77 (30.8%)	20 (36.4%)	13 (31.0%)	22 (22.2%)	16 (12.6%)	NA	
Post-menopause status (*N* = 682)	450 (66.0%)	180 (66.4%)	32 (57.1%)	23 (59.0%)	59 (60.2%)	92 (72.4%)	64 (70.3%)	0.19
Comorbidity^d^ (*N* = 666)	282 (42.3%)	108 (39.6%)	16 (28.6%)	14 (33.3%)	58 (58.6%)	56 (44.1%)	30 (43.5%)	0.00***
Tumour characteristics								
Ductal carcinoma *in situ* (*N* = 689)	144 (20.9%)	43 (15.8%)	8 (13.8%)	12 (28.6%)	19 (19.2%)	15 (11.8%)	47 (52.2%)	<0.001***
Invasive ductal carcinoma (*N* = 689)	495 (71.8%)	190 (69.6%)	40 (69.0%)	28 (66.7%)	84 (84.8%)	102 (80.3%)	51 (56.7%)	<0.001***
Invasive lobular carcinoma (*N* = 688)	66 (9.59%)	19 (6.96%)	10 (17.2%)	6 (14.3%)	6 (6.06%)	10 (7.87%)	15 (16.9%)	0.01**
Other carcinoma (*N* = 689)	32 (4.64%)	21 (7.69%)	0 (0.00%)	3 (7.14%)	0 (0.00%)	0 (0.00%)	8 (8.89%)	<0.001***
Positive oestrogen receptor status (*N* = 656)	568 (86.6%)	238 (87.2%)	48 (82.8%)	38 (90.5%)	80 (83.3%)	99 (84.6%)	65 (92.9%)	0.42
Positive progesterone receptor status (*N* = 655)	509 (77.7%)	221 (81.0%)	39 (68.4%)	32 (76.2%)	73 (76.0%)	82 (70.1%)	62 (88.6%)	0.02**
Positive HER2 receptor status (*N* = 674)	75 (11.1%)	23 (8.46%)	5 (8.62%)	7 (16.7%)	20 (20.8%)	16 (13.7%)	4 (4.49%)	<0.001***
Treatment characteristics								
Surgery (*N* = 688)								<0.001***
BCS	482 (70.1%)	214 (78.7%)	36 (63.2%)	29 (69.0%)	61 (61.6%)	81 (63.8%)	61 (67.0%)	
BCS with mammoplasty	33 (4.80%)	4 (1.47%)	0 (0.00%)	0 (0.00%)	0 (0.00%)	29 (22.8%)	0 (0.00%)	
Mastectomy without immediate reconstruction	67 (9.74%)	11 (4.04%)	11 (19.3%)	3 (7.14%)	17 (17.2%)	15 (11.8%)	10 (11.0%)	
Mastectomy with immediate reconstruction	106 (15.4%)	43 (15.8%)	10 (17.5%)	10 (23.8%)	21 (21.2%)	2 (1.57%)	20 (22.0%)	
Surgery to axilla (*N* = 686)								<0.001***
None	58 (8.45%)	27 (9.89%)	6 (10.5%)	4 (9.52%)	0 (0.00%)	6 (4.72%)	15 (17.0%)	
Sentinel lymph node biopsy	335 (48.8%)	198 (72.5%)	39 (68.4%)	24 (57.1%)	23 (23.2%)	0 (0.00%)	51 (58.0%)	
Axillary clearance	133 (19.4%)	48 (17.6%)	11 (19.3%)	13 (31.0%)	18 (18.2%)	22 (17.3%)	21 (23.9%)	
Axillary sampling	160 (23.3%)	0 (0.00%)	1 (1.75%)	1 (2.38%)	58 (58.6%)	99 (78.0%)	1 (1.14%)	
Radiotherapy (*N* = 690)	567 (82.2%)	239 (87.5%)	42 (72.4%)	39 (92.9%)	66 (66.7%)	118 (92.9%)	63 (69.2%)	<0.001***
Chemotherapy (*N* = 690)	248 (35.9%)	88 (32.2%)	22 (37.9%)	20 (47.6%)	43 (43.4%)	50 (39.4%)	25 (27.5%)	0.07
Hormonal therapy (*N* = 690)	563 (81.6%)	231 (84.6%)	51 (87.9%)	37 (88.1%)	81 (81.8%)	97 (76.4%)	66 (72.5%)	0.04**
Targeted therapy (*N* = 678)	66 (9.73%)	19 (7.01%)	5 (8.77%)	7 (20.6%)	18 (18.2%)	14 (11.0%)	3 (3.33%)	0.00***

BCS, breast cancer surgery; NA, not applicable.

a Percentages calculated excluding missing data.

b *P*-value corresponding to the χ^2^ test.

c Site F was excluded due to missing information.

d Comorbidity has been defined as a binary variable equal to 1 if patient presents a comorbidity.

** *P* < 0.00,

*** *P* < 0.01.

### Regression analysis

**Figure 1:**
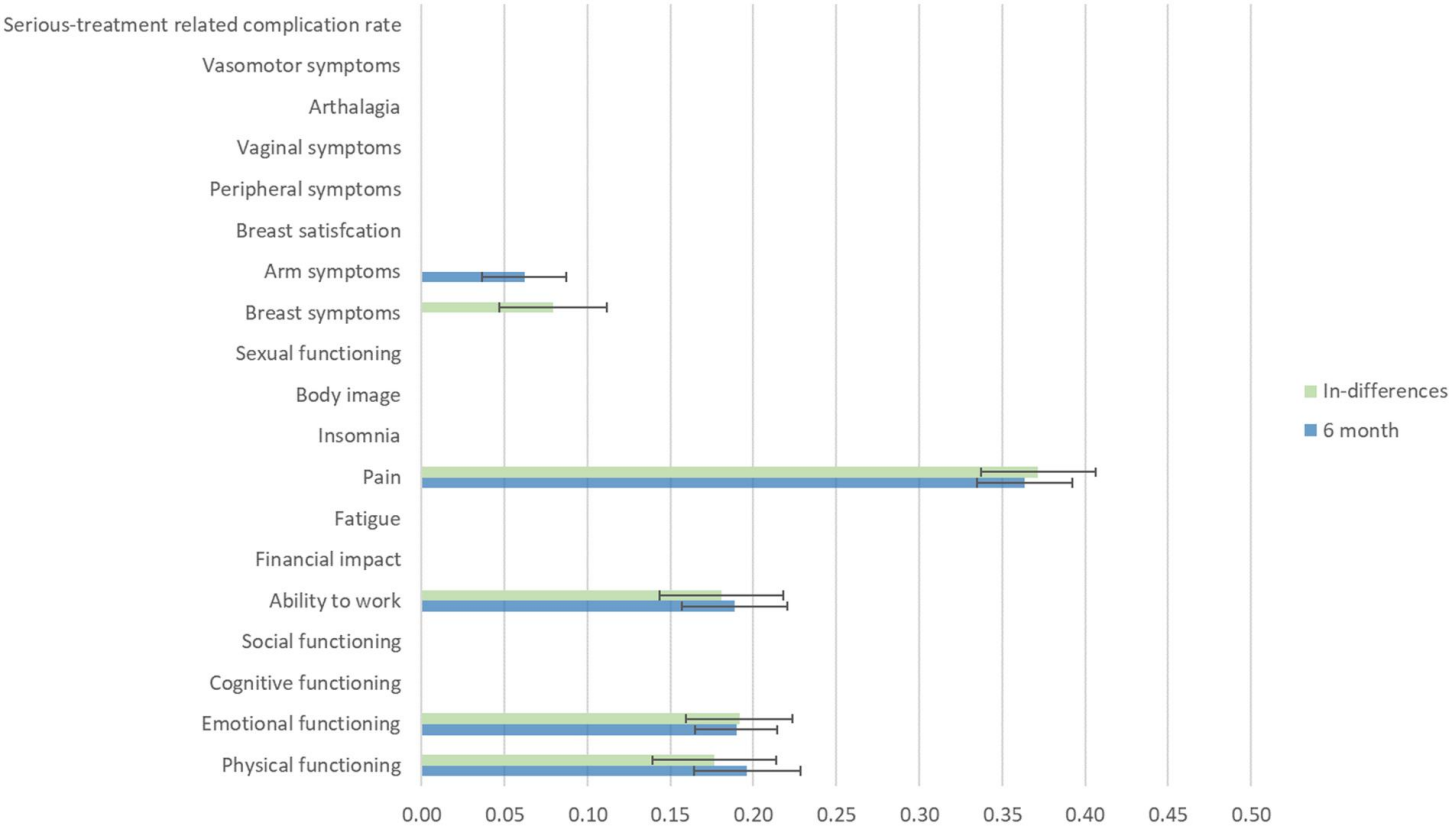
Patient-centred outcomes weights.

Regression results using the 6-month data showed five statistically significant PCO indicators, all of them being PROs. *Pain* showed as the PCO with the highest weight [36.3% (33.6–44.9%)] followed by *physical functioning* [19.6% (14.9–27.3%)], *emotional functioning* [19.0% (15.7–25.4%)], and *ability to work* [18.9% (14.1–26.6%)]. Results exploiting the variables in-differences also showed five PCOs, all of which were PROs. *Pain* showed as the PCO with the highest weight [37.2% (35.7–49.3%)] followed by *emotional functioning* [19.2% (15.6–28.1%)], *ability to work* [18.1% (13.3–28.0%)], and *physical functioning* [17.7% (12.9–27.5%)]. Figure 1 shows the PCOs weights. Table 2 shows the regression results as well as the PCOs weights.

**Table 2:** Regression results and PCOs weights.

	6 Months				in-differences			
	Beta (95% CI)	Beta standardized	*t*-value [Pr(>|t|)]	Weight	Beta (95% CI)	Beta standardized	*t*-value [Pr(>|t|)]	Weight
Physical functioning	0.002 (0.001 to 0.003)	0.212	6.61 [8.75E−11]**	0.196	0.002 (0.001 to 0.002)	0.202	5.44 [8.81E−08]**	0.177
Emotional functioning	0.002 (0.001 to 0.002)	0.205	8.27 [9.33E−16]**	0.190	0.001 (0.001 to 0.002)	0.219	6.85 [2.43E−11]**	0.192
Ability to work	0.001 (0.001 to 0.002)	0.204	6.38 [3.60E−10]**	0.189	0.001 (0.001 to 0.002)	0.207	5.53 [5.44E−08]**	0.181
Pain^a^	0.003 (0.002 to 0.003)	0.393	13.64 [8.00E−37]**	0.363	0.002 (0.002 to 0.003)	0.426	12.28 [4.46E−30]**	0.372
Arm symptoms^a^	0.001 (0 to 0.001)	0.067	2.66 [8.12E−03]**	0.062	–			
Breast symptoms^a^	–				−0.001 (−0.001 to 0)	−0.091	2.82 [5.03]**	0.079
Cognitive functioning	–				–			
Social functioning								
Financial impact^a^								
Fatigue^a^								
Insomnia^a^								
Body image								
Sexual functioning								
Breast satisfaction								
Vaginal symptoms^b^								
Arthralgia^c^								
Vasomotor symptoms								
Peripheral symptoms^a^								
Serious treatment-related complications rate								
Adjusted *R*^2^	0.716				0.571			

–, no statistically significant effects found; **p-value<0.05; CI, confidence interval.

a Higher scores for responses indicate worst health.

b Score ranges from 0 to 20.

c Score ranges from 0 to 4.

### Sensitivity analysis

**Figure 2:**
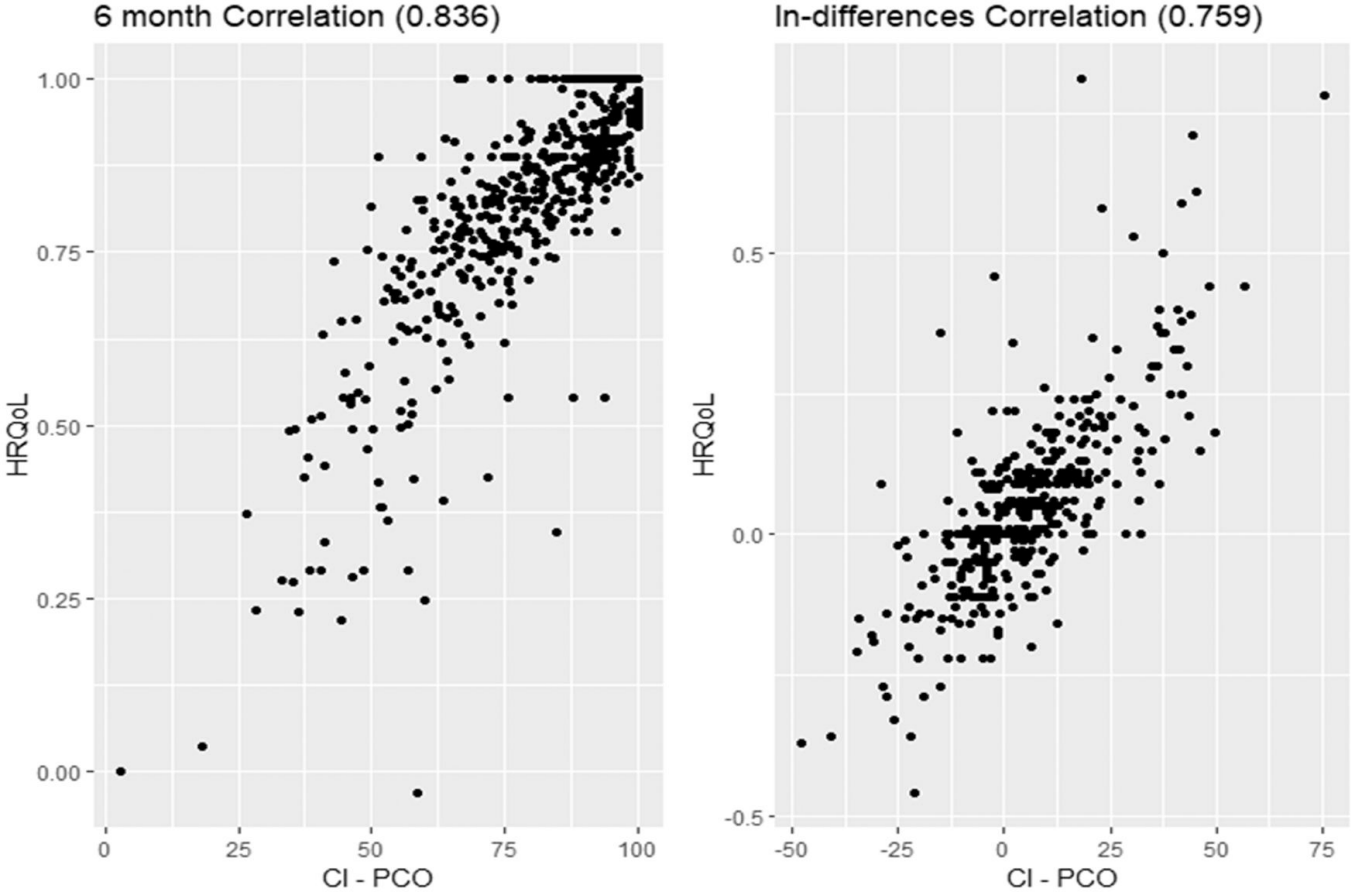
CI-PCO and HRQoL scatterplot.

The correlations between the CI-PCO and the HRQoL values at 6 months (ρ = 0.83) and in-difference (ρ = 0.76), all showed positive trends, as expected. In more depth, patients with a HRQoL value equal to 1 were assigned a variety of CI-PCO values distinguishing different levels among those with a very high HRQoL. Figure 2 shows a scatterplot of the CI-PCO and the HRQoL values at 6 months and in-differences.

A common pattern in weights between 6 months and in-differences based on four PCOs—*pain*, *ability to work*, *emotional functioning*, and *physical functioning*—presenting similar weights was found. *Arm symptoms* showed a statistically significant PCOs score at 6 months while *breast symptoms* did so at in-differences. Both PCOs also presented similar weights. A more in-depth assessment using the Mann–Whitney *U* test did not show statistically significant differences across the PCOs weights between both time structures.

## Discussion

Efforts to bring the VBHC into routine clinical practice is a current challenge in healthcare centres. One of the main VBHC challenges lies in combining health and economic outcomes using the value equation to reach one single VBHC figure. To the best of our knowledge, this is the first research attempting to quantitatively combine the PCOs of the value equation to reach a single numerator figure. This research might not only be of use in the breast cancer context but also for other medical conditions as a methodological pathway.

Combining multiple outcomes provides a simplified but holistic picture of patients’ health.^13^ Then, the numerator of the value equation was defined as a composite indicator of the PCOs^15^, a regression analysis was then undertaken to estimate the PCOs weights and consequently, the numerator of the value equation as a single figure was estimated.

In the context of healthcare, some authors have discussed the contribution of the VBHC approach to the so-called economic evaluation of healthcare interventions.^12^ They have underlined that the move to VBHC for assessing interventions should allow patient-centred frameworks to be incorporated. While the economic evaluation has used generic measurement instruments providing a measurement feasibility detrimental to individual sensitivity, the VBHC might complement the economic evaluation by adding a condition-specific and a more patient-centred approach beyond its utility as a standard value measure. In this paper, the regression analysis allowed the estimate of the PCOs weights, exploring the relationship between PCOs and HRQoL. HRQoL was used as a proxy of the health outcome to be measured in the numerator of the value equation.^16^ Although the use of a single measure such as the HRQoL might not enable development of a composite indicator,^17^ regression analysis might be a useful tool placing the patient at the centre of healthcare, estimating the corresponding PCOs weights, and consequently, providing a deeper understanding of the patient’s health status,^15^ according to the VBHC approach. The regression analysis showed a common pattern of PCOs with significant impact on HRQoL. These results were shown to be robust to sensitivity analysis conducted on time structures. Furthermore, the correlations between estimated CI-PCO and HRQoL values at 6 months and in-differences showed both positive trends, as expected. In more depth, Fig. 2 presents a scatterplot showing where uniform distribution of HRQoL values equal to 1 was associated with a distribution of a variety of CI-PCO values. Following the issues raised by Muldur,^17^ this illustrates that the CI-PCO—a more specific and multidimensional score than the HRQoL—might be more accurate and informative than a generic measure such as the HRQoL to assess the numerator of the value equation.

In a previous work, Schöner et al.^13^ compared three weighting methods to combine PROs into one composite measure: equal weighting, differential weighting, and principal component analysis (PCA). Beyond the *naïve* method of equal weighting, with the intention of exploiting the VOICE dataset, differential weighting was discarded in this paper. However, PCA was assessed to be included in the sensitivity analysis of this research, yet the low correlation among the PCOs did not allow this approach to be followed (see Supplementary Table S2). From a narrow perspective, normalization of PCOs might be the easiest solution; however, this would assume additive and linear measurement instruments. In this paper, the use of the regression analysis attempts to overcome these assumptions by avoiding manipulation of weights through *ad hoc* restrictions. Despite considering only 5 out of 17 PCOs proposed by the ICHOM Breast Cancer Group^6^^,^^7^ to estimate the numerator of the value equation as a composite indicator, this approach is not intended to call into question the ICHOM standard set nor the use of their corresponding PCOs. The ICHOM guidelines^6^^,^^7^ have been proved to guide routine clinical practice towards what really matters to patients,^18^ and to provide benchmarking on many indicators to identify best practices.^19^ This paper aims to move forward in VBHC addressing the estimation of PCOs weights to disentangle the figure of the numerator of the value equation, defined as a multidimensional measure underpinned by a composite indicator, exploiting a European dataset under regression analysis. To the best of our knowledge, this is the first research to combine the PCOs proposed by ICHOM to provide a single figure in the numerator of the value equation. This figure shows a step forward in VBHC to reach a holistic benchmarking across healthcare centres and a value-based payment. This research might also be applied in other medical conditions as a methodological pathway.

This study is not free of limitations. The lack of collected patient-reported experiences (PREs) in this research is considered an important limitation since PREs should be included in the PCOs. In particular, PROs were followed up at 6 months. This short follow-up in the context of breast cancer might not be considered as the entire patient pathway for some patients. Regarding the methodological approach, the main limitation is using HRQoL as a proxy of health outcome. Additionally, it is noted that the HRQoL was collected using the instrument EQ-5D-3L^8^ in only one out of the six healthcare centres—and was mapped from the EORTC QLQ-C30^11^ instrument for the other healthcare centres; however, it is noted that the patients who had HRQoL directly collected comprised almost 40% of the cohort.

## Supplementary Material

ckae060_Supplementary_Data

## Data Availability

The data underlying this article cannot be shared publicly due to privacy of individuals who participated in the study.
