# Supplementary material for: Disentangling the value equation: a step forward in value-based healthcare
Source: Eur J Public Health. 2024 Jun 14;34(4):632–8. doi: 10.1093/eurpub/ckae060 (PMC11293829; doi:10.1093/eurpub/ckae060)
Supplement: ckae060_Supplementary_Data [file ckae060_supplementary_data.pdf]

Tabla S1. Statistical descriptive. PROs at baseline and 6-month.

	Baseline (N=635)	Month 6 (N=616)	p-value
HRQoL	87·7 (12·3)	84·3 (16·8)	<0·001*
Physical functioning	91·9 (13·7)	83·7 (18·7)	<0·001*
Emotional functioning	70·0 (21·1)	80·4 (20·3)	<0·001*
Cognitive functioning	89·2 (16·7)	86·9 (19·9)	0·029*
Social functioning	90·7 (17·4)	88·7 (19·7)	0·062
Ability to work	92·3 (17·1)	83·2 (24·1)	<0·001*
Financial impact[a]	4·06 (15·1)	9·13 (22·2)	<0·001*
Fatigue[a]	15·9 (20·5)	28·0 (25·3)	<0·001*
Pain[a]	15·6 (18·7)	22·8 (24·4)	<0·001*
Insomnia[a]	32·2 (31·1)	32·7 (30·8)	0·795
Body image	92·9 (13·9)	84·8 (23·1)	<0·001*
Sexual functioning	22·5 (25·5)	20·7 (24·4)	0·247
Breast symptoms[a]	13·1 (15·3)	14·6 (15·7)	0·08
Arm symptoms[a]	8·36 (15·1)	12·2 (16·8)	<0·001*
Breast satisfcation	57·6 (16·2)	58·8 (13·3)	0·221
Peripheral symptoms[a]	10·9 (21·3)	19·6 (27·9)	<0·001*
Vaginal symptoms[b]	18·7 (1·90)	18·3 (2·36)	0·001
Arthalagia[c]	3·14 (1·08)	2·71 (1·27)	<0·001*
Vasomotor symptoms	13·2 (23·8)	35·1 (35·6)	<0·001*

HRQoL: Health-Related Quality of Life; PRO: Patient-Related Outcomes
[a]Higher scores for responses indicate worst health

[b]Score ranges from 0 to 20
[c]Score ranges from 0 to 4

Table S2. Pearson correlation matrix

	Baseline																		
	**HRQoL**	**physicalf**	**emotional**	**cognitive**	**social**	**labour**	**economic**	**fatigue**	**pain**	**insomnia**	**body**	**sexual**	**mama**	**arm**	**mama**	**peripheral**	**vaginal**	**arthalagia**	**vasos**
**HRQoL**	1·00																		
**Physical funct·**	0·64	1·00																	
**Emotional funct·**	0·42	0·10	1·00																
**Cognitive funct·**	0·50	0·38	0·40	1·00															
**Social functioning**	0·33	0·19	0·31	0·35	1·00														
**Ability to work**	0·61	0·58	0·18	0·40	0·35	1·00													
**Financial impact**	-0·14	-0·08	-0·09	-0·17	-0·11	-0·12	1·00												
**Fatigue**	-0·54	-0·56	-0·29	-0·53	-0·24	-0·51	0·21	1·00											
**Pain**	-0·67	-0·51	-0·22	-0·35	-0·26	-0·41	0·16	0·50	1·00										
**Insomnia**	-0·28	-0·14	-0·52	-0·29	-0·13	-0·13	0·10	0·34	0·16	1·00									
**Body image**	0·28	0·23	0·24	0·30	0·18	0·30	-0·05	-0·37	-0·15	-0·20	1·00								
**Sexual functioning**	0·29	0·29	0·07	0·17	0·14	0·16	-0·04	-0·16	-0·27	0·02	0·04	1·00							
**Breast symptoms**	-0·35	-0·16	-0·26	-0·28	-0·22	-0·32	0·09	0·26	0·35	0·15	-0·10	-0·05	1·00						
**Arm symptoms**	-0·39	-0·27	-0·21	-0·28	-0·25	-0·36	0·16	0·34	0·47	0·22	-0·13	-0·14	0·36	1·00					
**Breast satisfaction**	0·14	0·18	0·07	0·19	0·14	0·19	0·01	-0·30	-0·13	-0·06	0·27	0·11	-0·04	-0·08	1·00				
**Peripheral sympt·**	-0·33	-0·28	-0·14	-0·24	-0·07	-0·23	0·12	0·24	0·27	0·18	-0·17	-0·19	0·21	0·24	-0·04	1·00			
**Vaginal symptoms**	-0·09	-0·03	-0·01	0·09	0·06	0·03	0·01	-0·05	0·03	0·05	0·10	-0·09	-0·08	-0·07	0·07	-0·04	1·00		
**Arthralgia**	0·50	0·45	0·15	0·29	0·13	0·33	-0·14	-0·39	-0·54	-0·12	0·14	0·24	-0·18	-0·35	0·09	-0·30	-0·04	1·00	
**Vasomotor sympt·**	-0·05	-0·04	-0·06	-0·07	-0·05	-0·05	0·15	0·10	0·14	0·11	-0·11	-0·01	0·10	0·23	-0·04	0·17	-0·13	-0·14	1·00
	**6-month**																		

**HRQoL**	1·00																		
**Physical funct·**	0·65	1·00																	
**Emotional funct·**	0·45	0·29	1·00																
**Cognitive funct·**	0·30	0·26	0·47	1·00															
**Social functioning**	0·38	0·27	0·36	0·36	1·00														
**Ability to work**	0·67	0·68	0·28	0·27	0·43	1·00													
**Financial impact**	-0·17	-0·12	-0·22	-0·17	-0·26	-0·21	1·00												
**Fatigue**	-0·57	-0·62	-0·46	-0·41	-0·42	-0·57	0·30	1·00											
**Pain**	-0·69	-0·46	-0·26	-0·22	-0·32	-0·51	0·17	0·49	1·00										
**Insomnia**	-0·30	-0·27	-0·44	-0·23	-0·22	-0·28	0·21	0·35	0·23	1·00									
**Body image**	0·33	0·22	0·42	0·32	0·42	0·28	-0·28	-0·33	-0·32	-0·24	1·00								
**Sexual functioning**	0·20	0·27	0·19	0·17	0·16	0·21	-0·06	-0·20	-0·10	-0·06	0·08	1·00							
**Breast symptoms**	-0·28	-0·10	-0·14	-0·16	-0·21	-0·16	0·17	0·21	0·40	0·13	-0·21	0·08	1·00						
**Arm symptoms**	-0·41	-0·30	-0·24	-0·17	-0·19	-0·31	0·15	0·19	0·39	0·20	-0·27	-0·09	0·34	1·00					
**Breast satisfaction**	0·24	0·22	0·23	0·17	0·21	0·18	-0·13	-0·07	-0·13	-0·16	0·38	0·05	-0·14	-0·29	1·00				
**Peripheral sympt·**	-0·19	-0·14	-0·12	-0·22	-0·17	-0·12	0·10	0·25	0·26	0·16	-0·19	-0·12	0·09	0·21	-0·12	1·00			
**Vaginal symptoms**	0·03	-0·09	0·04	0·12	0·06	-0·05	0·04	-0·04	-0·06	-0·05	0·14	-0·08	-0·10	0·04	-0·04	-0·06	1·00		
**Arthralgia**	0·39	0·37	0·28	0·27	0·19	0·26	-0·09	-0·40	-0·40	-0·21	0·18	0·17	-0·16	-0·29	0·05	-0·23	0·06	1·00	
**Vasomotor sympt·**	-0·14	-0·06	-0·16	-0·17	-0·26	-0·13	0·23	0·15	0·18	0·22	-0·29	-0·04	0·21	0·21	-0·10	0·12	-0·18	-0·16	1·00

HRQoL: Health-Related Quality of Life
